# Supplementary material for: MetaRibo-Seq measures translation in microbiomes
Source: Nat Commun. 2020 Jun 29;11:3268. doi: 10.1038/s41467-020-17081-z (PMC7324362; doi:10.1038/s41467-020-17081-z)
Supplement: Supplementary file 10 — Supplementary Data 7 [file 41467_2020_17081_MOESM10_ESM.zip › File2/Confidence_VeryHigh_Taxonomy/307440_out.krona.html]

Javascript must be enabled to view this page.

members
magnitude
magnitudeUnassigned
count
unassigned
taxon
rank

307440\_out

13

2
superkingdom
13

13
phylum
976

13
class
200643

order
13
171549

13
family
171552

838
13
genus

13
species

SRS014415\_contig\_number\_1884SRS015590\_contig\_number\_contig-100\_3.135021SRS019285\_contig\_number\_contig-100\_18.21908SRS043841\_contig\_number\_10453SRS044535\_contig\_number\_14850SRS046502\_contig\_number\_6431SRS047433\_contig\_number\_24207SRS051610\_contig\_number\_contig-100\_2923.60750SRS065397\_contig\_number\_contig-100\_2.96434SRS077641\_contig\_number\_20010SRS104327\_contig\_number\_7353SRS143181\_contig\_number\_10355SRS148253\_contig\_number\_13495
165179
